# Targeting Senescence: A Review of Senolytics and Senomorphics in Anti-Aging Interventions

**DOI:** 10.3390/biom15060860

**Published:** 2025-06-13

**Authors:** Timur Saliev, Prim B. Singh

**Affiliations:** 1 Institute of Fundamental and Applied Medical Research, S.D. Asfendiyarov Kazakh National Medical University, Tole Bi Street 94, Almaty 050000, Kazakhstan; 2 School of Medicine, Nazarbayev University, Astana 010000, Kazakhstan; prim.singh@nu.edu.kz

**Keywords:** cellular senescence, senolytics, senomorphics, aging, senescence-associated secretory phenotype (SASP), anti-aging therapy, longevity

## Abstract

Cellular senescence is a fundamental mechanism in aging, marked by irreversible growth arrest and diverse functional changes, including, but not limited to, the development of a senescence-associated secretory phenotype (SASP). While transient senescence contributes to beneficial processes such as tissue repair and tumor suppression, the persistent accumulation of senescent cells is implicated in tissue dysfunction, chronic inflammation, and age-related diseases. Notably, the SASP can exert both pro-inflammatory and immunosuppressive effects, depending on cell type, tissue context, and temporal dynamics, particularly in early stages where it may be profibrotic and immunomodulatory. Recent advances in senotherapeutics have led to two principal strategies for targeting senescent cells: senolytics, which selectively induce their apoptosis, and senomorphics, which modulate deleterious aspects of the senescence phenotype, including the SASP, without removing the cells. This review critically examines the molecular mechanisms, therapeutic agents, and clinical potential of both approaches in the context of anti-aging interventions. We discuss major classes of senolytics, such as tyrosine kinase inhibitors, BCL-2 family inhibitors, and natural polyphenols, alongside senomorphics including mTOR and JAK inhibitors, rapalogs, and epigenetic modulators. Additionally, we explore the biological heterogeneity of senescent cells, challenges in developing specific biomarkers, and the dualistic role of senescence in physiological versus pathological states. The review also highlights emerging tools, such as targeted delivery systems, multi-omics integration, and AI-assisted drug discovery, which are advancing precision geroscience and shaping future anti-aging strategies.

## 1. Introduction

Aging is an inevitable biological process characterized by a progressive decline in physiological integrity, leading to impaired function and increased vulnerability to death. Among the various cellular and molecular mechanisms that contribute to aging, cellular senescence has emerged as a fundamental driver of age-related dysfunction and chronic disease [1,2]. Initially described as a mechanism to prevent the proliferation of damaged cells, senescence involves a state of stable cell cycle arrest coupled with profound changes in gene expression, morphology, and function. While this process plays a critical role in tissue remodeling, wound healing, and tumor suppression, the chronic accumulation of senescent cells contributes to a pro-inflammatory tissue environment and promotes aging-related pathologies.

Senescent cells secrete a variety of bioactive molecules collectively known as the senescence-associated secretory phenotype (SASP), which includes pro-inflammatory cytokines, chemokines, growth factors, and matrix-remodeling enzymes [3,4]. These factors have a dual nature: although they can be beneficial in short-term physiological contexts, such as promoting tissue repair or halting the proliferation of precancerous cells, their long-term persistence fosters a deleterious milieu that disrupts tissue homeostasis, impairs stem cell function, and promotes chronic inflammation and fibrosis [5]. The SASP is now recognized as a key mediator of the “inflammaging” process, a low-grade, chronic inflammation associated with aging, and has been implicated in a broad spectrum of diseases, including osteoarthritis, atherosclerosis, neurodegeneration, diabetes, and cancer [6,7].

In recent years, the concept of targeting cellular senescence has gained traction as a promising therapeutic strategy to delay aging and extend health span. Two major classes of anti-senescence therapies have emerged: senolytics, which selectively induce apoptosis in senescent cells, and senomorphics, which suppress the harmful effects of the SASP without killing the cells [8,9]. These interventions aim to reduce the burden of senescent cells and modulate their secretory activity, thereby alleviating tissue dysfunction and enhancing resilience against stress and disease. Encouragingly, preclinical studies in mouse models have demonstrated that clearing senescent cells can improve tissue function, delay the onset of age-related diseases, and even extend lifespan [10]. These findings have spurred a surge in translational research, with multiple clinical trials now underway to evaluate the safety and efficacy of senescence-targeting agents in humans [11,12,13].

Senescent cells are key contributors to aging, marked by irreversible growth arrest and often the secretion of pro-inflammatory factors known as the senescence-associated secretory phenotype (SASP). While transient senescence aids in tissue repair and tumor suppression, chronic accumulation of senescent cells promotes tissue dysfunction, inflammation, and age-related decline. Kondratyeva et al. (2025) demonstrated that treatment with growth differentiation factor 11 (GDF11) partially reversed the senescent phenotype in mesenchymal stem cells (MSCs), improving viability and angiogenic function [14]. This highlights the impact of senescence on stem cell aging and the potential of rejuvenation therapies.

In the context of skin aging, Wyles et al. (2024) [15] showed that topical platelet-derived exosomes reduced senescence markers (p16^INK4a^, p21^CIP1^) and SASP components, improving skin structure, supporting the idea that local clearance or modulation of senescent cells can restore tissue function. At the systemic level, Nishizawa et al. [16] found that the transcription factor BACH1 suppresses senescence through ferroptosis-induced secretion of FGF21, which improved metabolism and extended lifespan in progeria models. This points to a non-cell-autonomous role of senescent cells in aging regulation. These studies affirm that senescent cells are not passive bystanders but active drivers of aging. They impair tissue regeneration, induce chronic inflammation, and influence systemic health. Therapeutic strategies that target senescent cells, via senolytics, senomorphics, or rejuvenation signals like GDF11 and FGF21, hold promise for mitigating age-related decline and extending health span. Emerging evidence strongly supports the therapeutic potential of eliminating or modulating senescent cells to enhance healthspan and delay age-related decline. Senescent cells accumulate with age and contribute to chronic inflammation, tissue dysfunction, and age-related diseases. Poblocka et al. (2021) developed a targeted antibody–drug conjugate (ADC) that selectively eliminates senescent cells by binding to β2-microglobulin (B2M), a surface marker enriched in senescence [17]. The ADC delivers a cytotoxic payload specifically to senescent cells, reducing their burden without affecting healthy tissue. This approach offers a promising, selective senolytic strategy. In contrast, Zumerle et al. (2024) used a natural polyphenol extract [18], Haenkenium (HK), from Salvia haenkei to reduce senescence markers and improve physical condition in aged mice. One active compound, luteolin, modulates the p16–CDK6 interaction, suppressing senescence-associated pathways without killing the cells. This senomorphic strategy demonstrates how modulating, rather than clearing, senescent cells can improve healthspan. Dorronsoro et al. [19] showed that extracellular vesicles (EVs) from young mesenchymal stem cells reduced cellular senescence and extended health span in mice, offering a regenerative, cell-free alternative with low risk and high translational potential. Together, these studies highlight that both senolytics and senomorphics can mitigate the harmful effects of senescent cells, offering viable strategies to improve tissue function and extend health span [20]. Despite the promise of these interventions, several challenges remain. The heterogeneity of senescent cell populations, the context-dependent effects of senescence, and the potential off-target consequences of eliminating or altering senescent cells raise concerns about the safety and specificity of current approaches [21]. Moreover, the lack of robust biomarkers to identify and monitor senescent cells in vivo complicates both research and clinical application [22]. A deeper understanding of the molecular mechanisms governing senescence, improved methods for detecting senescent cells, and the development of next-generation therapeutics with greater selectivity and minimal side effects will be critical to unlocking the full potential of this field [21,23]. 

As we continue to explore the complex biology of cellular senescence and refine our strategies for intervening in this process, targeting senescence holds significant promise not only for mitigating the effects of aging but also for transforming the way we approach the prevention and treatment of age-related diseases [24]. This review provides an overview of current strategies and advances in senolytics and senomorphics, examines their therapeutic potential and limitations, and discusses future directions in this rapidly evolving field of geroscience.

## 2. Senolytics: Eliminating Senescent Cells

Senolytics are a rapidly advancing class of therapeutic agents designed to selectively eliminate senescent cells, which accumulate with age and contribute to chronic inflammation, tissue degeneration, and age-related diseases [25,26]. Cellular senescence is a state of irreversible cell cycle arrest triggered by various stressors such as telomere shortening, DNA damage, mitochondrial dysfunction, and oncogene activation [27]. While transient senescence has beneficial roles in development, wound healing, and tumor suppression, the chronic persistence of senescent cells in aged tissues has been implicated in the pathogenesis of numerous conditions, including osteoarthritis, cardiovascular disease, pulmonary fibrosis, and neurodegeneration.

Senescent cells evade immune clearance by activating anti-apoptotic signaling pathways, often termed senescent cell anti-apoptotic pathways (SCAPs), which differentiate them from their healthy counterparts [28]. Senolytic agents exploit this vulnerability by disabling these pro-survival mechanisms, thereby triggering apoptosis selectively in senescent cells while sparing non-senescent tissue [29,30]. The first generation of senolytic therapies emerged with the identification of dasatinib, a tyrosine kinase inhibitor, and quercetin, a flavonoid that inhibits PI3K and other kinases [31] (Table 1). These two compounds, used in combination (D + Q), were found to clear senescent pre-adipocytes and endothelial cells in mouse models [32]. In aged or diseased animals, intermittent administration of D + Q improved physical function, reduced senescent cell burden, and in some cases, extended lifespan. These findings laid the groundwork for clinical trials in humans, where early results suggest improvements in physical performance and reductions in senescence biomarkers in patients with idiopathic pulmonary fibrosis and diabetic kidney disease.

**Table 1:** Comparative Overview of Major Senolytic Classes.

Senolytic Class	Molecular Targets	Represent Agents	Mode of Action	Strengths	Limitations/Challenges	Ref.
Tyrosine Kinase Inhibitors	Src family kinases, Eph receptors	Dasatinib	Inhibits pro-survival tyrosine kinases upregulated in certain SnC types	Effective in senescent preadipocytes and progenitors	Cell-type specificity; potential for systemic toxicity	[33]
Flavonoid Polyphenols	PI3K/AKT, NF-κB, ROS pathways	Quercetin, Fisetin	Induces apoptosis via oxidative stress and suppression of anti-apoptotic signaling	Low toxicity; orally bioavailable; broad applicability	Variable potency; poor bioavailability in vivo	[34]
BCL-2 Family Inhibitors	BCL-2, BCL-xL, BCL-w	Navitoclax (ABT-263), ABT-737	Blocks anti-apoptotic proteins, sensitizing SnCs to apoptosis	Potent and broad-acting across senescent phenotypes	Thrombocytopenia due to BCL-xL inhibition in platelets	[35]
FOXO4-p53 Disruptors	FOXO4-p53 complex	FOXO4-DRI peptide	Disrupts nuclear retention of p53, restoring apoptotic signaling	Selective SnC clearance; rejuvenates aged tissues	Peptide delivery limitations; currently preclinical	[36]
HSP90 Inhibitors	Heat shock protein 90	17-DMAG, Geldanamycin derivatives	Destabilizes chaperone-dependent survival proteins in SnCs	Targets multiple stress response pathways	General cytotoxicity; lacks SnC specificity	[37]
CDK/p53 Pathway Modulators	MDM2-p53, CDK4/6	UBX0101, Nutlin-3	Modulates cell cycle regulators and apoptotic checkpoints	Targeted for local intra-articular applications	Short half-life; mixed clinical trial results	[38]
Natural Senolytics (Plant-Derived)	ROS generation, NF-κB, SASP factors	Piperlongumine, Curcumin analogues	Promotes redox imbalance and downregulates SASP-related pathways	Low-cost, multi-targeted; dietary sources	Low potency; unclear pharmacokinetics and dosing strategies	[39]

Building on these foundational discoveries, researchers have developed a diverse range of senolytic agents targeting different aspects of senescent cell biology. One key area of focus is the BCL-2 family of proteins, which are upregulated in senescent cells to resist apoptosis. Navitoclax (ABT-263), an inhibitor of BCL-2 and BCL-XL, has demonstrated potent senolytic activity across several tissues [35,40] (Table 1). However, it has been limited in clinical translation by dose-dependent thrombocytopenia, as platelets also rely on BCL-XL for survival [41]. Similar agents, such as ABT-737, have shown promise in preclinical settings but share similar safety concerns [42]. A structurally related compound, ABT-737, was an earlier-generation BCL-2 family inhibitor that similarly targets BCL-2, BCL-XL, and BCL-w. ABT-737 has shown promising senolytic effects in preclinical models, particularly in the clearance of senescent cells from irradiated tissues and in models of pulmonary fibrosis and atherosclerosis [43]. However, ABT-737, like navitoclax, has demonstrated hematologic toxicity in vivo, including thrombocytopenia and neutropenia, due to the on-target depletion of non-senescent cells that rely on BCL-2/XL signaling for survival. This safety profile has limited its direct clinical use and prompted efforts to either develop selective delivery systems (e.g., nanoparticle carriers or conjugation with senescence-targeted ligands) or to identify next-generation BCL-2 inhibitors with improved senescent cell specificity.

Furthermore, both ABT-263 and ABT-737 underscore a broader challenge in senolytic drug development: the molecular overlap between survival pathways in senescent and healthy cells, especially in high-turnover tissues. As a result, recent research is focusing on transient, intermittent dosing strategies, combination regimens with senomorphics, or targeting downstream effectors unique to senescence, to mitigate adverse effects.

Another strategy targets the interaction between FOXO4 and p53, which plays a role in maintaining the survival of senescent cells. The FOXO4-DRI peptide disrupts this interaction, reactivating p53-mediated apoptosis and eliminating senescent cells in various models [44]. HSP90 inhibitors, such as 17-DMAG, destabilize chaperone-mediated survival pathways in senescent cells, offering another route for clearance [45,46]. More recently, UBX0101 and related molecules have been designed to modulate the p53-MDM2 axis and CDK activity to selectively eliminate senescent chondrocytes in osteoarthritic joints, although clinical progress has been mixed [38].

In addition to synthetic compounds, several naturally occurring molecules have demonstrated senolytic potential. Fisetin, a dietary flavonoid found in fruits such as strawberries and apples, is particularly notable for its broad efficacy across different senescent cell types [47,48]. It reduces systemic inflammation, improves tissue homeostasis, and extends health span in mouse models. Fisetin is currently being tested in several human trials for its potential to alleviate age-associated dysfunction. Other natural agents like piperlongumine and synthetic curcumin derivatives are also under investigation, offering potentially safer and more accessible alternatives to synthetic drugs [49,50].

Despite these promising advances, the development of senolytics faces several key challenges. Many compounds lack cell-type specificity, raising concerns about off-target effects and toxicity in non-senescent tissues. Some, like navitoclax, induce significant side effects at effective doses. Moreover, the heterogeneity of senescent cells, both within and across tissues, means that a one-size-fits-all senolytic is unlikely to exist. Optimization of dosing schedules, such as intermittent “hit-and-run” regimens, is being explored to limit toxicity while maintaining efficacy. Another major limitation is the absence of robust, non-invasive biomarkers for senescent cell burden in humans, which impairs the ability to stratify patients or monitor treatment response. Furthermore, there is concern that simply removing senescent cells without addressing the underlying damage or inflammatory microenvironment may lead to their rapid re-accumulation.

Nonetheless, the translational momentum in this field is strong. Several senolytic compounds are in early-phase clinical trials for diseases including Alzheimer’s disease, chronic kidney disease, frailty, and osteoarthritis [51]. New approaches are being investigated to improve targeting and minimize off-target effects, including the use of antibody-drug conjugates, nanoparticles, and tissue-specific prodrugs. In the future, senolytic therapies may be combined with senomorphics, regenerative interventions, or immune modulators to enhance both efficacy and tissue repair. As our understanding of cellular senescence deepens, senolytics stand at the forefront of a potential paradigm shift in aging and chronic disease treatment, with the promise of not only extending lifespan but improving health span through selective cellular rejuvenation. 

While senolytics represent a promising class of interventions to delay aging and mitigate age-related diseases, their clinical development is complicated by the heterogeneous nature of cellular senescence across different tissues. Senescence is not a uniform process; it varies significantly depending on cell type, tissue context, and developmental stage [52]. For example, senescent fibroblasts in dermal tissue may exhibit distinct SASPs and survival pathways compared to senescent endothelial cells in vascular tissue or chondrocytes in the joint [53]. Likewise, the function and fate of senescent cells can differ: in some settings, they are immunosuppressive and pro-fibrotic, while in others, they may promote inflammation or contribute to tissue repair.

This context dependency has direct implications for senolytic therapies, which are often designed based on generalized senescence mechanisms, such as the upregulation of BCL-2 family proteins or the FOXO4-p53 axis. However, given the variability in survival pathways between senescent cell types, senolytic agents may be effective in one tissue but ineffective, or even harmful, in another. For example, navitoclax and ABT-737 demonstrate robust senolytic activity in certain cell populations but cause thrombocytopenia due to BCL-XL dependence in platelets, highlighting the risk of unintended toxicity in non-senescent cells [35,54].

To overcome these challenges, future senolytic strategies will likely need to adopt organ-specific or cell-type-specific targeting approaches. This may involve the use of tissue-specific promoters, targeted delivery vectors (e.g., nanoparticles or antibody–drug conjugates), or prodrug strategies activated by tissue-enriched enzymes. These precision delivery systems could enhance the selective clearance of senescent cells in target tissues while sparing others.

Moreover, age and disease stage may influence the composition and behavior of senescent cells, particularly in organs with active regeneration or developmental plasticity. For instance, the response to senolytics in aged skeletal muscle stem cells may differ markedly from that in senescent astrocytes in neurodegeneration. Tailoring therapeutic timing and delivery to the developmental stage, disease context, and regenerative capacity will be crucial for optimizing outcomes.

Additionally, combinatorial approaches, such as pairing senolytics with senomorphics, anti-inflammatory agents, or regenerative therapies, may help address the complex interplay between senescent cells and their microenvironment. These strategies could mitigate tissue-specific rebound effects or functional deficits that occur following widespread cell clearance.

## 3. Senomorphics: Modulating the SASP

**Figure 1:**
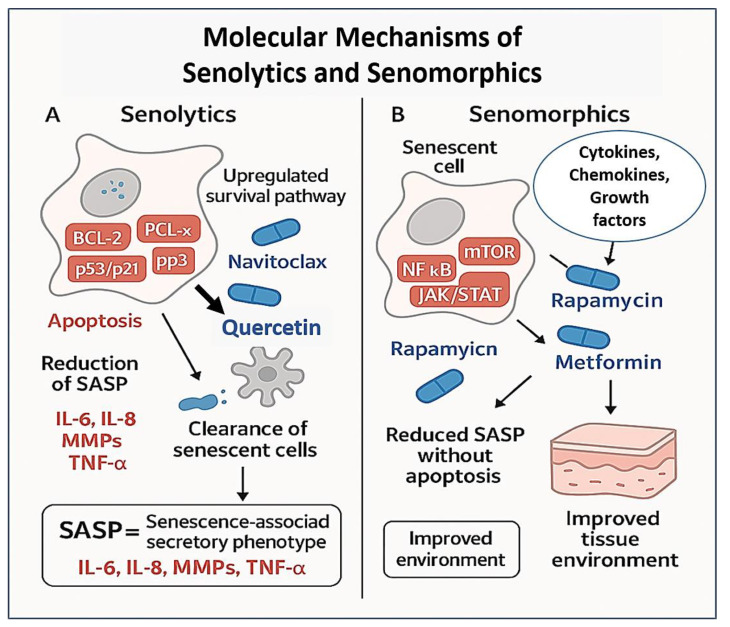
Molecular mechanisms of senolytics and senomorphics in anti-aging interventions.

While senolytics aim to eliminate senescent cells from aging tissues, an alternative therapeutic approach, known as senomorphics, seeks instead to modulate the phenotype and secretory behavior of senescent cells without necessarily inducing their apoptosis [55,56] (Figure 1).

This class of interventions is also frequently referred to as senostatics in the literature [57,58]. While the terms are often used interchangeably, some authors draw a subtle distinction: “senomorphics” emphasize the modulation of the senescent phenotype, particularly the suppression or alteration of the senescence-associated secretory phenotype (SASP), whereas “senostatics” highlight the stabilization of senescent cells in a non-harmful, quiescent state without promoting further senescence propagation. In practice, however, both terms describe agents that mitigate the detrimental effects of senescent cells without eliminating them, and they are commonly treated as synonyms in the current field.

The strategy of eliminating senescent cells from aging tissues holds great promise but may be inappropriate in certain physiological contexts, particularly in tissues where senescent cells play beneficial roles in processes such as wound healing, tissue remodeling, or immune surveillance. In such cases, senomorphics offer a more refined approach by targeting and suppressing the pro-inflammatory and tissue-damaging components of the senescence-associated secretory phenotype (SASP). The SASP comprises a complex and dynamic mixture of cytokines, chemokines, growth factors, matrix metalloproteinases (MMPs), and bioactive lipids secreted by senescent cells, which contribute to chronic inflammation and tissue dysfunction when left unchecked [13,59]. Persistent SASP signaling is now widely recognized as a key mediator of chronic inflammation in aging (“inflammaging”), as well as a driver of paracrine senescence, tumor promotion, and tissue degeneration.

The SASP is regulated by several stress-responsive transcription factors and intracellular signaling pathways, including NF-κB, C/EBPβ, mTOR, JAK/STAT, and p38 MAPK [60]. Targeting these pathways has led to the identification of various senomorphic agents capable of attenuating SASP expression and its downstream pathological effects (Table 2). Among the most studied are mTOR inhibitors such as rapamycin and its analogs (rapalogs), which can dampen SASP production by interfering with the translation of IL-1α, a key upstream driver of the SASP cascade [61,62]. In preclinical models, rapamycin has been shown to suppress senescence-related inflammation, improve stem cell function, and extend lifespan, without necessarily inducing cell death [63,64]. Its oral bioavailability and known safety profile from clinical use in transplantation and oncology make it one of the most promising senomorphic agents under evaluation for geroprotective interventions.

Another major class of senomorphics includes JAK inhibitors, such as ruxolitinib and baricitinib, which block downstream inflammatory signaling pathways induced by SASP components like IL-6 and IL-8 [65,66] (Table 2). In both mouse models and early human studies, JAK inhibition has reduced systemic inflammation, improved hematopoietic function, and restored tissue homeostasis in aged organs [67,68]. These agents are especially promising in chronic inflammatory conditions associated with clonal hematopoiesis and myeloid skewing. Importantly, senomorphics like JAK inhibitors offer a potentially safer long-term strategy compared to senolytics, particularly for chronic dosing in older adults, since they avoid the risks associated with cell ablation and regeneration imbalance [69,70].

**Table 2:** Overview of Major Senomorphic Agents in Aging and Disease Modulation.

Agent/Class	Primary Targets	Mechanism of Action	Current Status	Key Notes	Ref.
Rapamycin (Sirolimus)	mTORC1	Inhibits mTOR-mediated translation of SASP factors (e.g., IL-1α)	Multiple trials in aging (e.g., NCT02432287)	Shown to improve immune function, reduce inflammation	[71]
Ruxolitinib/Baricitinib	JAK1/2	Blocks JAK-STAT signaling, suppressing IL-6/IL-8 mediated SASP amplification	Phase 2 trials for frailty, inflammation	Also used for myelofibrosis and rheumatoid arthritis	[72]
p38 MAPK Inhibitors	p38 MAPK	Reduces SASP via inhibition of upstream inflammatory signaling	Preclinical/early clinical	Reduces IL-6, TNF-α production in senescent cells	[73]
BET Inhibitors	BRD4, chromatin modifiers	Represses transcription of SASP-related genes by reducing enhancer/promoter activity	Preclinical	Emerging class with epigenetic modulation potential	[74]
NF-κB Inhibitors	NF-κB pathway (IKK complex)	Blocks transcription of pro-inflammatory cytokines central to the SASP	Mostly preclinical	Non-specific immunosuppression is a challenge	[75]
Glucocorticoids (e.g., Dexamethasone)	Glucocorticoid receptor/NF-κB	Represses SASP cytokine expression, suppresses general inflammation	Clinically approved; repurposing debated	Broad-spectrum effects; not ideal for long-term use	[76]
Metformin	AMPK/NF-κB/mTOR	Indirectly suppresses SASP by activating AMPK, inhibiting mTOR, and dampening NF-κB	Widely used; TAME trial (NCT04245771) ongoing	Mild SASP modulation; favorable safety profile	[77]
Resveratrol	SIRT1/NF-κB	Activates SIRT1, inhibits NF-κB signaling and oxidative stress	Nutraceutical; limited clinical trials	Low potency; bioavailability limitations	[78]
HDAC Inhibitors	Histone deacetylases	Alters chromatin accessibility of inflammatory gene promoters	Preclinical/repurposing from oncology	Potential for selective SASP modulation	[79]

In addition, glucocorticoids, long used for their immunosuppressive effects, exhibit some senomorphic properties by downregulating SASP cytokine transcription through inhibition of NF-κB [80,81]. However, their broad-spectrum immunosuppression and significant side-effect profile limit their suitability for aging interventions. More targeted NF-κB inhibitors are under investigation, aiming to selectively block SASP-associated gene transcription while preserving beneficial immune function. Other experimental approaches include p38 MAPK inhibitors, which interfere with upstream signaling of SASP activation, and BET bromodomain inhibitors, which suppress SASP gene expression by modifying chromatin accessibility at pro-inflammatory loci [82].

Notably, the functional heterogeneity of the SASP presents both an opportunity and a challenge. While many SASP components are pro-inflammatory and deleterious, some elements are involved in tissue regeneration, immune surveillance, and fibrosis resolution. Therefore, future senomorphic strategies must aim for selective SASP modulation, suppressing harmful secretions while preserving or even enhancing beneficial paracrine signals [83]. Precision targeting of SASP regulators, possibly through AI-guided compound design or tissue-specific delivery systems, could offer a path forward in fine-tuning this complex phenotype [84].

By selectively modulating key components of the senescence-associated secretory phenotype, such as pro-inflammatory cytokines (e.g., IL-6, IL-1β), matrix metalloproteinases, and chemokines, it may be possible to preserve the beneficial functions of senescent cells, including tissue repair and tumor suppression, while minimizing their deleterious pro-aging effects. Advances in artificial intelligence and machine learning can accelerate the identification of small molecules or biologics that target specific SASP pathways, with greater predictive accuracy and reduced off-target toxicity. Simultaneously, innovations in nano-medicine and targeted delivery platforms, such as lipid nanoparticles, antibody-drug conjugates, or cell-penetrating peptides, are enabling the localization of senomorphic therapies to affected tissues or cell populations. This precision approach may help overcome the heterogeneity of senescent cell populations and the context-dependent nature of SASP, thereby reducing systemic side effects and improving therapeutic efficacy. Ultimately, such strategies could enable dynamic, tunable modulation of senescence-related inflammation, paving the way for more refined and personalized anti-aging interventions.

An important and increasingly recognized aspect of the senescence-associated secretory phenotype (SASP) is its time-dependent nature, which significantly influences its biological effects. The composition and functional consequences of the SASP evolve dynamically over time following the onset of senescence, and this temporal heterogeneity plays a crucial role in determining whether the SASP exerts beneficial or detrimental outcomes [85,86].

In the early stages of senescence, particularly during acute tissue responses or following oncogene activation, the SASP can exhibit pro-fibrotic and immunosuppressive characteristics. Early SASP components may include transforming growth factor-beta (TGF-β), vascular endothelial growth factor (VEGF), and various extracellular matrix (ECM) proteins, which promote fibrosis and tissue remodeling while concurrently suppressing certain immune responses [87]. In this context, the SASP may help maintain tissue integrity and dampen potentially excessive immune activation, an effect that can be protective in the short term. However, this transient immunosuppression may also create a local environment that allows for immune evasion, tissue scarring, or even tumor progression if senescent cells are not efficiently cleared [88].

As senescence becomes chronic and unresolved, the SASP transitions to a more pro-inflammatory profile, marked by the upregulation of interleukins such as IL-6, IL-8, and IL-1β, as well as matrix metalloproteinases (MMPs) [89]. These factors promote immune cell recruitment, stem cell dysfunction, and tissue degeneration. This shift contributes to the pathological remodeling of tissues, persistent inflammation, and the propagation of senescence to neighboring cells via paracrine signaling.

Understanding the temporal evolution of the SASP is therefore essential when designing therapeutic interventions. For instance, efforts to modulate the SASP (e.g., via senomorphics) should consider not only which SASP components to suppress but also when during the senescence timeline such modulation will be most effective or least disruptive to physiological repair mechanisms [90]. Therapeutic strategies that blunt early pro-fibrotic or immunosuppressive SASP elements without impairing beneficial acute functions may offer the most precise and context-sensitive benefits.

Senomorphics may also complement senolytic therapy in combinatorial regimens. For instance, transient senomorphic pre-treatment could suppress SASP-induced systemic toxicity before senolytic-induced cell clearance, or conversely, senomorphics could be administered post-senolysis to suppress SASP release from surviving or neighboring cells [21,91]. This dual-pronged approach is being actively explored in preclinical models, especially in the context of chemotherapy-induced senescence, metabolic dysfunction, and neuroinflammation.

As the field advances, a deeper mechanistic understanding of SASP regulation, alongside improved in vivo biomarkers of SASP burden, will be essential for rational drug development. Moreover, since the SASP varies depending on tissue context, senescence trigger, and duration, the concept of “context-specific senomorphism” may guide the design of next-generation interventions. In sum, while senolytics represent a bold approach to cellular rejuvenation, senomorphics offer a nuanced and adaptable strategy, one that may hold particular promise for the chronic management of age-related inflammation, frailty, and organ dysfunction without the risks associated with broad cellular depletion. 

## 4. Challenges and Limitations

Despite growing enthusiasm and remarkable progress in the field of senescence-targeting therapies, numerous scientific and clinical challenges remain that hinder the broad implementation of senolytics and senomorphics in aging and disease-modifying interventions. A central obstacle is the inherent heterogeneity of senescent cells across tissues, time, and triggering stimuli. Senescence is not a single, uniform phenotype; instead, it is a context-dependent program influenced by cell type, senescence inducer, micro-environmental signals, and organismal age [92]. As a result, senescent cells exhibit varying dependencies on survival pathways, distinct SASP profiles, and divergent sensitivities to pharmacological agents. This diversity complicates the development of “universal” senolytic or senomorphic drugs and increases the risk of inconsistent efficacy across patient populations or disease models.

Equally limiting is the lack of specific, non-invasive biomarkers that can reliably identify senescent cell burden in vivo (Table 3). While markers such as p16^INK4a^, p21^CIP1^, SA-β-gal activity, and DNA damage foci (γH2AX) are widely used in experimental settings, they are not exclusive to senescence and are often inaccessible in clinical tissues. Furthermore, no consensus exists on how to quantify systemic senescence load or monitor therapeutic response dynamically. This gap in biomarker development impedes clinical translation by making patient selection, efficacy tracking, and dose optimization difficult in human trials. Without validated biomarkers, it is also challenging to distinguish beneficial, transient senescence (such as that involved in wound repair) from chronic, deleterious senescence driving pathology.

**Table 3:** Challenges, Implications, and Possible Solutions for Senescence-Targeting Therapies.

Challenge	Description	Implications	Possible Solutions
Heterogeneity of Senescent Cells	Senescent cell features vary by tissue, trigger, and aging context	Limits development of one-size-fits-all senotherapeutics; variable drug response	Use tissue-specific profiling (e.g., single-cell omics); design context-dependent or combinatorial therapies
Lack of Specific Biomarkers	No robust, non-invasive markers to quantify senescent cell burden in vivo	Difficult to identify target patients, track efficacy, or determine optimal dosing	Develop circulating biomarkers, imaging tracers, and senescence-specific transcriptomic signatures
Safety Concerns of Senolytics	Off-target effects on non-senescent cells (e.g., platelets, immune cells)	Heightened toxicity risk, especially in frail elderly patients	Engineer targeted delivery systems (e.g., nanoparticles, prodrugs, ADCs); explore intermittent “hit-and-run” dosing
Limitations of Senomorphics	Broad-acting effects, immunomodulation, lack of clearance	Potential SASP rebound; unclear long-term benefits	Develop pathway-selective senomorphics; combine with senolytics or regenerative therapies
Context-Dependent Role of Senescence	Senescence contributes positively to tissue repair and tumor suppression in some settings	Risk of unintended tissue damage or impaired regeneration if senescent cells are eliminated indiscriminately	Adopt adaptive modulation strategies; tailor timing and duration of intervention to specific physiological contexts
Regulatory and Ethical Uncertainty	Aging is not a recognized medical indication; endpoints and trial designs lack standardization	Slows approval and integration into mainstream care; public skepticism	Define aging-related surrogate endpoints; develop ethical guidelines for preventive gerotherapeutics

The safety profile of senolytic agents represents another critical concern (Table 3). While senolytics aim to selectively eliminate senescent cells, many current compounds exhibit off-target effects, potentially harming non-senescent cells or vital immune populations [93]. For example, BCL-2 family inhibitors like navitoclax, although effective in clearing senescent cells, are associated with dose-limiting thrombocytopenia due to BCL-xL inhibition in platelets [94]. Similarly, broad-acting kinase inhibitors and pro-apoptotic agents can induce systemic toxicity, particularly when used chronically or in combination with other therapeutics [95]. This risk is especially pronounced in aged individuals, who are the primary target population but also the most vulnerable to adverse events due to multimorbidity, frailty, and polypharmacy. As such, improving the selectivity and delivery of senotherapeutics, potentially through tissue-targeted prodrugs, antibody–drug conjugates, or nanoparticle-based systems, remains an urgent priority.

In the case of senomorphics, while they offer a potentially safer approach by modulating rather than eliminating senescent cells, they present their own challenges (Table 3). Most senomorphic compounds exhibit pleiotropic effects, acting on multiple pathways beyond the SASP. This broad mechanism of action increases the risk of unintended consequences, including suppression of beneficial immune responses or interference with cellular stress responses required for tissue integrity. Moreover, long-term administration of senomorphics may create pharmacodynamic tolerance, necessitating escalating doses or combination therapies [96]. The fact that senomorphics do not remove senescent cells also raises the possibility of a rebound in SASP production upon treatment cessation, or continued accumulation of dysfunctional cells, potentially compromising long-term efficacy.

From a translational perspective, one of the most underappreciated barriers is the contextual role of senescence in physiology. Senescence has evolved as a beneficial program in certain settings, including embryogenesis, tissue repair, and tumor suppression. Therefore, indiscriminate suppression or clearance of senescent cells may impair regenerative responses, disrupt tissue homeostasis, or increase cancer risk in certain contexts. For example, in the skin or liver, transient senescence can coordinate repair processes via SASP-mediated recruitment of immune and progenitor cells. Removing these cells prematurely could lead to impaired healing or excessive fibrosis [97]. Understanding the temporal dynamics and reversibility of senescence is essential for ensuring that interventions are not only effective but also aligned with tissue-specific physiological needs.

Regulatory and ethical frameworks for senotherapeutics remain underdeveloped, reflecting the novelty and complexity of targeting aging biology. Unlike traditional therapeutics designed to treat acute or well-defined diseases, senotherapeutics are increasingly being proposed for preventive use to delay or mitigate age-related functional decline in largely asymptomatic individuals. This shift challenges conventional paradigms of clinical trial design, including the selection of endpoints, risk–benefit assessment, and duration of follow-up. The absence of standardized criteria for measuring aging outcomes, such as biological age, functional capacity, or resilience, further complicates regulatory evaluation. Without clear definitions of what constitutes clinical “success” in the context of aging interventions, trials may yield ambiguous results that are difficult to interpret or compare across studies.

To address these issues, a multi-stakeholder approach is urgently needed. Regulatory bodies such as the FDA and EMA should collaborate with geroscience researchers to define age-related surrogate endpoints that are biologically meaningful, clinically actionable, and acceptable for conditional approval. Longitudinal cohorts, biomarker validation studies, and real-world evidence must be leveraged to establish reliable predictors of therapeutic efficacy and long-term safety. Adaptive trial designs, including umbrella and platform trials, may also offer greater flexibility to test multiple agents or dosing strategies in aging populations.

Another critical barrier is the influence of public perception and commercial hype. The growing market for unregulated anti-aging products and exaggerated claims around longevity-enhancing interventions threatens to erode public trust and scientific integrity. To prevent this, stricter oversight of marketing practices, coupled with public education initiatives, is essential. Medical societies and public health institutions should play a central role in communicating the current evidence base, distinguishing between experimental and validated therapies, and ensuring informed decision-making by consumers.

Economic and logistical considerations further complicate the large-scale implementation of senotherapeutics. Aging affects virtually all individuals, implying that successful therapies could generate enormous demand across populations and age groups. This raises concerns about healthcare system capacity, affordability, and equitable access. To address these challenges, health technology assessments (HTAs) should be employed early in drug development to evaluate cost-effectiveness and inform pricing strategies. Moreover, targeting high-risk groups, such as individuals with multi-morbidity, frailty, or accelerated biological aging, may provide the most immediate and measurable benefits, enabling a phased and prioritized rollout of senescence-targeting interventions.

In summary, realizing the clinical potential of senotherapeutics will require more than scientific progress; it will demand regulatory innovation, ethical foresight, public engagement, and strategic health system planning. By addressing these interconnected challenges, the field can move toward the responsible, effective, and equitable integration of anti-aging therapies into mainstream medical care. These span biological uncertainty, technological limitations, clinical safety, and translational logistics. Addressing these barriers will require interdisciplinary collaboration, rigorous biomarker discovery, smarter drug delivery platforms, ethical oversight, and a cautious yet forward-thinking approach to clinical trial design. Only through these efforts can senotherapeutics fulfill their potential as transformative tools for extending health span and mitigating age-related disease burden. 

## 5. Future Directions

Looking ahead, the future of senescence-targeting therapies lies in moving beyond proof-of-concept studies toward clinically actionable, safe, and personalized interventions that can meaningfully extend the human health span. As our understanding of cellular senescence becomes more nuanced, so too must our therapeutic strategies evolve, from blunt cell elimination to context-sensitive modulation, from broad-acting compounds to precision medicine, and from isolated interventions to integrative, systems-level approaches. The next generation of senotherapeutics will likely combine molecular specificity, spatiotemporal control, and functional synergy with other hallmarks of aging to achieve durable rejuvenation without compromising physiological senescence functions [98].

**Figure 2:**
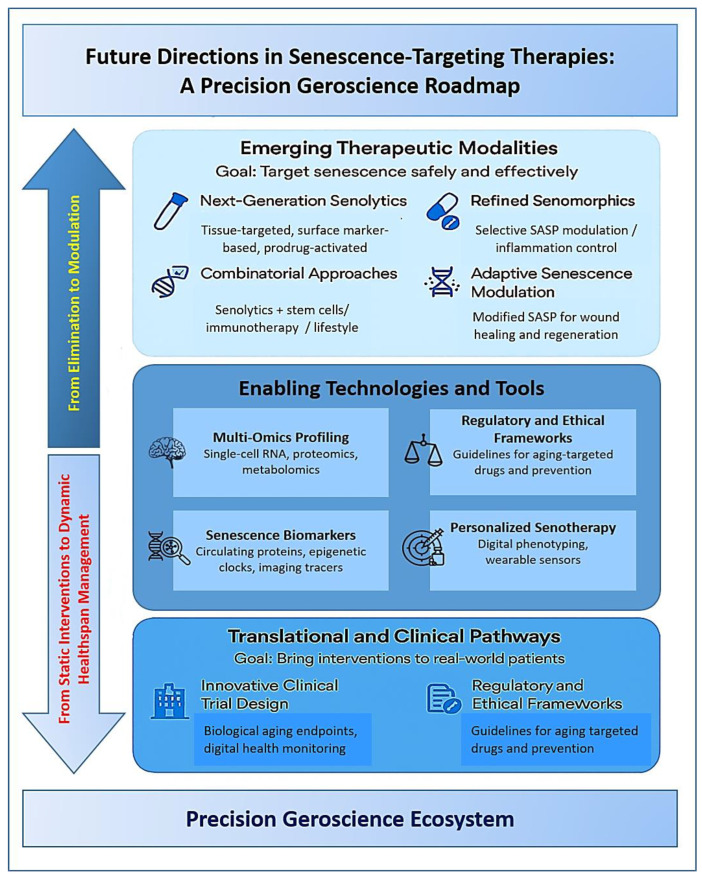
Future Directions in Senescence-Targeting Therapies: A Precision Geroscience Roadmap.

A key frontier in this evolution is the development of next-generation senolytics and senomorphics with enhanced tissue targeting, reduced off-target toxicity, and improved pharmacokinetics (Figure 2). Efforts are underway to engineer senolytic prodrugs that become activated only in senescent cells based on unique metabolic signatures, pH gradients, or enzymatic profiles. Similarly, antibody-drug conjugates (ADCs) that recognize senescence-specific surface markers, such as DPP4, uPAR, or B2M, offer a means of delivering senolytics selectively to affected tissues while sparing healthy cells [99]. Nanoparticle-based delivery systems also hold promise for increasing the precision and local concentration of senotherapeutic agents, especially in hard-to-reach tissues like the brain or bone marrow [100,101]. These approaches not only improve safety profiles but may also allow for combinatorial targeting of different senescent cell populations within the same organism.

At the same time, multi-omics profiling and single-cell technologies are beginning to revolutionize how senescent cells are identified, classified, and understood. Advances in transcriptomics, epigenomics, proteomics, and metabolomics now enable high-resolution mapping of senescence heterogeneity across tissues and disease states (Figure 1). These datasets are facilitating the construction of senescence “atlases” that will guide biomarker discovery, therapeutic targeting, and patient stratification. Moreover, the integration of artificial intelligence and machine learning into omics analysis is accelerating the identification of senescence-specific pathways and predictive biomarkers [102]. These tools will be instrumental in overcoming one of the field’s greatest hurdles: the reliable, non-invasive detection and monitoring of senescent cell burden in humans.

In parallel, clinical trial design in geroscience must undergo innovation to accommodate the unique challenges of targeting aging biology. Traditional trial endpoints, such as disease incidence or short-term symptom resolution, may not fully capture the long-term benefits of senotherapeutics. Instead, surrogate endpoints like inflammatory biomarkers, composite frailty indices, epigenetic aging clocks, and digital mobility assessments may offer more sensitive and practical measures of therapeutic efficacy [103]. Adaptive trial designs, decentralized data collection, and real-world evidence frameworks could also help accelerate the regulatory approval process. Importantly, as interventions move from disease-specific indications toward preventive or health-span-enhancing applications, ethical and regulatory frameworks will need to be refined to ensure safety, equity, and accountability.

Beyond pharmacological strategies, combining senotherapeutics with regenerative medicine, immunotherapy, and lifestyle interventions may yield synergistic effects that restore tissue function while removing or suppressing cellular damage. For example, senolytic pre-treatment may improve the engraftment and efficacy of stem cell therapies by clearing the inflammatory milieu, while senomorphics may enhance the benefits of caloric restriction or exercise by reducing systemic SASP-driven inflammation [104]. The convergence of aging research with other cutting-edge domains, such as gene editing, autophagy modulation, and gut microbiome engineering, may unlock entirely new therapeutic paradigms for systemic rejuvenation.

A critical and increasingly recognized frontier in senotherapeutic development involves targeting not only senescent cells themselves but also the microenvironmental context in which they reside, specifically, the aging stem cell niche. Evidence from multiple organ systems demonstrates that senescence within the niche profoundly influences tissue homeostasis, regenerative capacity, and response to therapy. In the neural stem cell niche of the subventricular zone, for instance, senescent astrocytes and microglia disrupt neurogenic signaling and foster a pro-inflammatory milieu that impairs neural regeneration. Similarly, in the hematopoietic stem cell (HSC) niche, age-associated changes in mesenchymal stromal cells, endothelial support cells, and the extracellular matrix reduce HSC self-renewal, skew lineage differentiation, and contribute to immunosenescence.

These insights underscore the need for niche-aware senotherapeutic strategies that address both senescent stem cells and the dysfunctional supportive cells that surround them. Precision senolytic or senomorphic interventions aimed at specific niche-resident populations, such as senescent glia in the CNS or aged stromal cells in the bone marrow, may help restore a pro-regenerative environment. Moreover, the use of localized delivery systems, including niche-targeted nanoparticles, hydrogels, or organ-specific prodrugs, can enhance therapeutic specificity and reduce systemic toxicity. Integrating spatial and biophysical mapping of stem cell niches into emerging senescence atlases, alongside single-cell multi-omics, will further refine our understanding of how senescence unfolds at the tissue level and guide the design of context-specific interventions.

Incorporating the niche perspective into senescence research and therapeutic development represents a vital step toward true precision geroscience. By addressing the organ-specific complexity of senescent cell interactions, future senotherapeutics will be better equipped to rejuvenate regenerative microenvironments and support long-term tissue repair, moving beyond cell elimination to holistic tissue revitalization.

Another promising direction is the concept of “adaptive senescence modulation”, which recognizes that not all senescent cells are harmful at all times. Emerging strategies aim to temporally tune senescence responses rather than simply suppress them. For instance, short-term SASP activation may be allowed or even induced to promote regeneration or immune recruitment, followed by controlled silencing to avoid chronic inflammation [83]. This more sophisticated approach to senescence biology will require intelligent biomaterials, feedback-controlled drug delivery systems, and deeper integration of systems biology.

Looking further ahead, there is a growing interest in personalized senotherapy, tailored to an individual’s senescence profile, genetic background, and environmental exposures. Just as oncology has moved toward personalized medicine based on tumor subtyping and molecular diagnostics, senescence-targeting interventions may one day be guided by individual aging trajectories mapped through deep longitudinal phenotyping. This shift will depend on the development of robust clinical senescence diagnostics, wearable biosensors for early detection of functional decline, and data platforms that integrate lifestyle, physiological, and molecular aging data.

Ultimately, the future of senescence-targeting interventions lies not in a single molecule or magic bullet, but in a precision geoscience ecosystem, one that harmonizes molecular therapies, diagnostics, digital health, and public policy to compress morbidity, reduces age-related disease burden and enables more people to live longer, healthier, and more functional lives. Realizing this vision will require sustained investment, cross-disciplinary collaboration, and careful ethical stewardship, but the scientific foundation is rapidly solidifying. If successfully implemented, senotherapeutics could represent a defining advance of 21st-century medicine, shifting our healthcare paradigm from reactive disease treatment to proactive health preservation across the lifespan.

## 6. Conclusions

Targeting cellular senescence has emerged as one of the most compelling strategies for addressing the biological underpinnings of aging and age-related diseases. The development of senolytics and senomorphics offers complementary approaches, either by clearing harmful senescent cells or by modulating their pro-inflammatory and degenerative secretory profiles. Robust preclinical evidence supports the therapeutic potential of these agents in mitigating frailty, improving tissue function, and extending health span in diverse animal models.

However, the path to widespread clinical application is complex. Challenges such as the heterogeneity of senescent cells, the dual role of senescence in physiology and pathology, and the lack of specific in vivo biomarkers underscore the need for cautious and sophisticated intervention strategies. Safety concerns, particularly with senolytic agents, must be rigorously addressed through improved drug design, targeted delivery technologies, and personalized treatment paradigms.

Future directions in the field are likely to include the development of next-generation senotherapeutics with enhanced specificity and minimal toxicity, integration with regenerative medicine and lifestyle interventions, and the implementation of precision senotherapy guided by molecular profiling and AI-driven diagnostics. Additionally, the convergence of geroscience with other domains, such as immunotherapy, epigenetics, and systems biology, will be crucial in creating holistic approaches to aging and longevity. In conclusion, while the translation of senescence-targeting therapies into routine clinical practice remains in its early stages, the conceptual and technological foundation is rapidly strengthening. Senolytics and senomorphics are poised not only to redefine the management of chronic diseases but also to shift the healthcare paradigm from disease treatment to health preservation, heralding a new era in the science of aging.

## Data Availability

Not applicable.
